# The Human Semicircular Canals Orientation Is More Similar to the Bonobos than to the Chimpanzees

**DOI:** 10.1371/journal.pone.0093824

**Published:** 2014-04-07

**Authors:** Marwan El Khoury, José Braga, Jean Dumoncel, Javotte Nancy, Remi Esclassan, Frederic Vaysse

**Affiliations:** 1 University of Toulouse, Molecular Anthropology and Image Synthesis Laboratory (Centre National de la Recherche Scientifique), Toulouse, France; 2 University of Bordeaux, Faculty of dentistry, Bordeaux, France; Museo Nazionale Preistorico Etnografico ‘L. Pigorini’, Italy

## Abstract

For some traits, the human genome is more closely related to either the bonobo or the chimpanzee genome than they are to each other. Therefore, it becomes crucial to understand whether and how morphostructural differences between humans, chimpanzees and bonobos reflect the well known phylogeny. Here we comparatively investigated intra and extra labyrinthine semicircular canals orientation using 260 computed tomography scans of extant humans (*Homo sapiens*), bonobos (*Pan paniscus*) and chimpanzees (*Pan troglodytes*). Humans and bonobos proved more similarities between themselves than with chimpanzees. This finding did not fit with the well established chimpanzee – bonobo monophyly. One hypothesis was convergent evolution in which bonobos and humans produce independently similar phenotypes possibly in response to similar selective pressures that may be associated with postural adaptations. Another possibility was convergence following a “random walk” (Brownian motion) evolutionary model. A more parsimonious explanation was that the bonobo-human labyrinthine shared morphology more closely retained the ancestral condition with chimpanzees being subsequently derived. Finally, these results might be a consequence of genetic diversity and incomplete lineage sorting. The remarkable symmetry of the Semicircular Canals was the second major finding of this article with possible applications in taphonomy. It has the potential to investigate altered fossils, inferring the probability of post-mortem deformation which can lead to difficulties in understanding taxonomic variation, phylogenetic relationships, and functional morphology.

## Introduction

Phenotypic traits have been used for decades for the purpose of reconstructing the evolutionary history of humans (*Homo sapiens*) and their closest relatives [1], chimpanzee and bonobo species (*Pan troglodytes* and *Pan paniscus*, respectively). More recently, phenotypic data have been supplemented by growing evidence from the genome wide sequencing analysis [2]–[4].

The comparison between human and chimpanzee genomes revealed genetic differences accumulated since these species diverged from their common ancestor [5]. The hominin-*Pan* split date was lately recalibrated to at least 7–8 million years [6]. It is often argued that chimpanzee subspecies and bonobos carry no or marginal genetic differences, when compared to the corresponding differences seen in humans from different continents [7]–[9]. Besides, common chimpanzees show the greatest population stratification when compared to all other great ape lineages, while humans and western chimpanzees show a remarkable dearth of genetic diversity when compared to other great apes. It was also found that the rate of gene loss in the human branch is not different as compared to other internal branches in the great ape phylogeny [10]. Recently, more extensive comparisons revealed that bonobo and chimpanzee genomes were not necessarily more closely related to each other than to the human one [2].

The phenotype of bonobos has received less attention than that of chimpanzees, despite several studies investigating dental development [11] and morphology [12], [13], craniometry [14], [15], intralabyrinthine angles [16]–[19], cranial development and vascularization [20]–[22], endocranial ontogeny and morphology [23]. For some traits, bonobos appear to be less diverse than chimpanzees in both their phenotype [12], [14], [20], and DNA [9], [24]–[27]. Interestingly, the variation of some phenotypic traits has been shown to correlate more closely with genetic data than others [5].

For instance, the inner ear morphology has proven to be useful to assess diversity among extant and fossil primates [18], [28]. However, a few studies have focused on variability within the genus *Pan* and have compared such variations with the extant human figures [16], [18], [19], [29]. Studies from the early 70's [16]–[18] included large samples focused on angles taken from 2D radiographs. They concluded that the bonobo and the chimpanzee labyrinths are more similar to each other than to the human one.

Here, we investigated differences in the orientation of semicircular canals (SCC) starting from the null hypothesis (H0) that not only *Homo* and *Pan* differ in SCC orientation, but also that the two *Pan* species are more similar as compared to humans. Based on a sample of 260 medical X-ray computed tomography (CT) scans, we applied a mathematical model verified with microcomputed tomography (μCT) scans [30] and measurement error quantification. We subsequently investigated how these features discriminate the three species and we discussed our results in the context of their evolutionary relationships. Besides, we explored the existence of eventual sexual dimorphism related to human SCCs.

## Material and Methods

### Ethics statement

The human CT scans were provided from medical CT. The data reported here involved no experimentation on human subjects but only reprocessing of existing anonymized scan data. The use of these data for the present purpose was in respect of bioethical laws in France. Written consent was given by the patients for their information to be stored in the hospital database and used for research purpose. The “comité de protection des personnes – Bordeaux” (French IRB) approved the use of these data for the present purpose.

The *Pan* sample was composed of a set of inner ear reconstructed from dry skulls. We obtained permission from the Royal Museum of Central Africa of Tervuren (Belgium) and the Museum of Comparative Zoology at Harvard University (Cambridge, MA, USA) to access the collections. The collections were elaborated in the early twentieth century from mostly wild-shot animals donated to the museums. These collections were widely used in several studies including Lieberman et al. [31] and Durrleman et al. [23]. The samples were donated to the museums, and the parties involved in the hunting of the animals held the proper permits.

### Samples

Our sample was composed of 137 anonymized human clinical records (*H. sapiens*: 70 females and 67 males), 61 *P. paniscus* and 62 *P. troglodytes* (8 *P. troglodytes verus* and 54 *P. troglodytes schweinfurthi*) (see Table 1 and Table S1). The three species were represented by subadult and adult individuals.

**Table 1:** Description of the sample.

Class	abbreviation	Human	Bonobo	Chimpanzee	Apes	Total
infant	NJ1	1	3	7	10	11
infant stage 2	J1	0	11	5	16	16
young juvenile	J2	35	14	11	25	60
old juvenile	J3	50	10	10	20	70
sub-adult	A1	16	8	15	23	39
adult	A2	35	15	14	29	64
Total		137	61	62	123	260

Number of individuals according to Age classes [30] and species.

The human sample was composed of patients from the Pasteur Hospital (Toulouse, France) and the Faculty of Dentistry at the University of Toulouse (France), scanned between 2007 and 2010. These individuals had been referred for cranial trauma, inflammation of maxillary sinuses or neonatal distress but were found to be free of reportable abnormalities having any direct or indirect impact on inner ear morphology. The pixel size ranged from 0.3 to 0.49 mm and the slice thickness from 0.3 to 0.8 mm (for detailed information see Table S1). The human CT scans were provided from medical CT.

The *Pan* sample was composed of wild animals. The pixel size ranged from 0.27 to 0.49 mm and the slice thickness from 0.5 to 1 mm (for detailed information see Table S1).

The Maturational Status (MS) was assessed using dental stages [32] and reported in Table S1.

### Data collection

Data were saved initially as Digital Imaging and Communications in Medicine (DICOM) format files, and then as Tagged Image File Format (TIFF) files.

**Figure 1:**
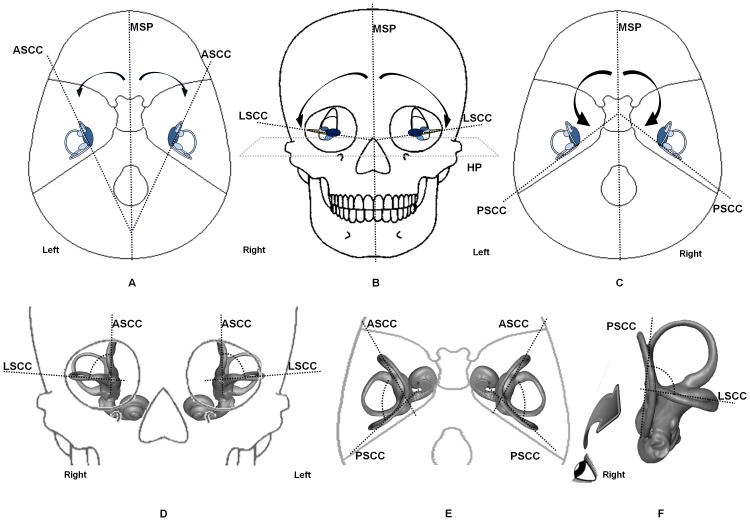
SCC angle representations. (a) MSP/ASCC angles. (b) Orientation of LSCC with MSP and HP (c) MSP/PSCC angles (d) ASCC/LSCC angles (e) ASCC/PSCC angles (f) LSCC/PSCC angles.

Thirty landmarks (Table 2) were placed on the CT images using Amira software to best represent each SCC, as well as the midsagittal (MSP) and horizontal (HP) planes of the skull, used as references (Table 3). Anterior, posterior and lateral SCCs were referred to as ASCC, PSCC and LSCC, respectively. Each SCC was represented by three landmarks located at the center of its lumen [33]. The vestibule was also represented by one single landmark (see Method S1 for detailed information). Each SCC plane coordinate was then calculated from its landmarks as well as from the vestibular one. Then we calculated the 3D angles between planes (Figure 1) using the dot product of the two plane normal vectors [30]. For the main results, significance level was set at p = 0.01.

**Table 2:** Definition of the landmarks used in the present study.

N°	Name	Definition	Bookstein landmarks type
1	Frontal crest (Fc)	Summit of the Fc	II
2	Crista galli (Cr)	Summit of Cr	I
3	Internal occipital crest (iOc)	Medial most eminent point on the iOc	II
4	Vomer (Vm)	Point on the posterior border of the Vm	II
5	Nasopalatine foramen (NPf)	Central point of the NPf	I
6	Foramen Magnum (fMo)	Midpoint on the anterior border of the fMo	II
7,8	Infraorbital foramina (IOf)	Midpoint of the IOf	I
9,10	Supraorbital foramen (SOf)	Cranial part of the notch of the SOf	II
11,12	Vestibule (Vb)	Center of the lumen of the Vb	I
13,16	ASCC (middle)	Superior-most point at the center of the ASCC lumen	II
14,17	ASCC (anterior)	Anterior point at the center of the ASCC lumen before its ampulla	II
15,18	ASCC (posterior)	Posterior point at the center of the ASCC lumen before the common crus	II
19,22	LSCC (anterior)	Anterior point at the center of the LSCC lumen before its ampulla	II
20,23	LSCC (middle)	Lateral-most point at the center of the LSCC lumen	II
21,24	LSCC (posterior)	Posterior point at the center of the LSCC lumen before joining the vestibule	II
25,28	PSCC (inferior)	Inferior point at the center of the PSCC lumen before its ampulla	II
26,29	PSCC Right (middle)	Posterior-lateral-most point at the center of the PSCC lumen	II
27,30	PSCC Right (superior)	Superior point at the center of the PSCC lumen before the common crus	II

Anterior, posterior and lateral SCCs (semicircular canals) were noted respectively ASCC, PSCC and LSCC. Landmarks of **Type I** were well defined locally; their homology from individual to another was strongly supported. **Type II** landmarks was corresponding to points which position was first defined locally using specific structures but it was also depending on less specific factors such as the maximum or minimum of a curve. When using type II landmarks the individual to individual homology was only supported geometrically, to calculate plane coordinates for example.

**Table 3:** Definition of the reference planes used in the study.

planes	landmarks (Table 2)
**Mid-Sagittal Plane**	MSP: 1, 2, 3, 4, 5, 6
**Horizontal Plane**	HP: 7, 8, 11, 12
**Semi Circular Canal planes**	ASCC right: 11, 13, 14, 15
	ASCC left: 12, 16, 17, 18
	LSCC right: 11, 19, 20, 21
	LSCC left: 12, 22, 23, 24
	PSCC right: 11, 25, 26, 27
	PSCC left: 12, 28, 29, 30

Anterior, posterior and lateral SCC (semicircular canals) were noted respectively ASCC, PSCC and LSCC.

Body size is important to consider when comparing the size of morphological structures between species. However it was not considered in our study as it has no real impact on angular measurements [19]. The radii of the SCC were not reported as they did not meet our validation criteria.

### Statistics

Statistical tests were performed with R software. Before angular comparisons, the normality was tested using either Shapiro-Wilk's test (N<50) or D'Agostino's test (N>50). The normal probability plot and frequency histogram were also established to visually check the normality of the distribution. The equality of variances was tested with Levene's tests.

The angular measurements were compared using a one-way ANOVA and Tukey's HSD post-hoc test providing a correction due to multiple comparisons. The Kruskall-Wallis test for multiple comparisons was used when normality criteria were not completed. A between-group principal component analysis (bgPCA) [34] of angular measurements was also applied on angles presented in Table 4. The bgPCA was previously successfully used to discriminate labyrinthine shape differences between predefine groups of chimpanzees or fossil hominins [35], [36]. Measurements were tested also for sexual dimorphism, fluctuating asymmetry, anti-symmetry and directional asymmetry [37], [38] using a two-way anova and student's t-tests.

**Table 4:** Statistical comparisons of the SCC orientation between humans, chimpanzees and bonobos.

	Humans	← p →	Chimpanzees	← p →	Bonobos	← p →	Humans
Canal pair	N = 137	H vs C	N = 62	C vs B	N = 61	B vs H	N = 137
ASCC/LSCC*	**74.2±4.4°**	<0.001	**77.0±7.4°**	NS	**74.8±7.1°**	NS	**74.2±4.4°**
ASCC/PSCC*	**111.2±6.4°**	<0.001	**105.7±7.6°**	<0.001	**109.5±6.1°**	NS	**111.2±6.4°**
LSCC/PSCC**	**88.2±6.2°**	<0.001	**80.1±10.8°**	<0.001	**86.9±10.5°**	NS	**88.2±6.2°**
LSCC right/left**	**19.7±8.9°**	NS	**22.0±9.5°**	NS	**21.3±15.2°**	NS	**19.7±8.9°**
ASCC right/PSCC left**	**10.6±5.4°**	<0.001	**19.2±9.5°**	<0.001	**12.8±7.8°**	NS	**10.6±5.4°**
ASCC left/PSCC right**	**11.4±6.4°**	<0.001	**18.0±10.6°**	<0.001	**14.3±9.2°**	NS	**11.4±6.4°**
MSP/ASCC*	**34.9±5.1°**	<0.001	**39.1±5.9°**	NS	**38.1±4.6°**	<0.001	**34.9±5.1°**
MSP/LSCC*	**80.9±5.5°**	NS	**80.6±6.6°**	NS	**82.9±8.6°**	NS	**80.9±5.5°**
MSP/PSCC*	**145.3±6.2°**	<0.001	**142.1±8.0°**	<0.001	**146.1±7.4°**	NS	**145.3±6.2°**
HP/LSCC*	**25.9±6.7°**	<0.001	**23.0±7.2°**	<0.001	**26.1±7.5°**	NS	**25.9±6.7°**

Angles measurements and angles comparisons between species showing subtle differences between humans and bonobos and marked differences with chimpanzees (NS  =  not significant, H =  humans, B =  Bonobos, C = Chimpanzees). Anterior, posterior and lateral SCC were referred to as ASCC, PSCC and LSCC. (*) parametric tests were used since angular measurements showed normal distribution. (**) non parametric tests were used since angular measurements did not show normal distribution; however parametric tests results were consistent with those of non parametric tests. All angles in degrees.

### Measurement error and validation

In order to validate the reproducibility of our results, we ran a test to assess the extent of intra-observer error. We used 60 randomly selected subjects. All measurements were taken twice by one observer (MEK), on separate days and without any knowledge on sex, age and species attributions [39]. Mean error was then analyzed using the angular mean error and a two-way ANOVA (sides*individuals) to compare Measurement Error relative to fluctuating asymmetry [40]. We did not find significant differences between the two sets of measurements over the 60 selected subjects (p>0.80) resulting in a mean error of 0.48°±0.30. ME was found to be significantly lower than fluctuating asymmetry (p<0.02).

Additionally, we ran other tests to validate the accuracy of angular calculation by using μCT as a comparison. To this end, Four *P. troglodytes* were scanned using both clinical Cone Beam Computed Tomography (K9500, Trophy, KODAK) and μCT (Xtreme CT by SCANCO - Switzerland). Non parametric Kruskal-Wallis tests were used to detect differences between conventional-CT and μCT groups. Since μCT images covered only the petrous bones, only inter-SCC angles were computed. No significant differences were detected between the two imaging procedures (p>0.63). Similar evidence resulted from an independent additional test run on three *Papio anubis*, and one *Gorilla gorilla* using the same procedure as the four *P. troglodytes.*

## Results

Medical CT images are contentious to measure very small dimensions [41] due to their spatial resolution [42]. The intra-observer error was found negligible in the present study and no statistical differences were found between the CT and μCT imaging procedures providing an indication of the method reliability [30].

### Intra-specific comparisons

The possible influence of sex was not assessed in *Pan*, as sex attribution was not available for all specimens. In humans, we found sexual dimorphism in ASCC/PSCC and LSCC/PSCC angles. Both of them were more open in females than in males (respectively +2.7°, p<0.001 and +2.9°, p<0.001). Previously, sexual dimorphism was already pointed out as reflecting allometry only [19].

No differences were found between MS confirming that SCC orientations undergo no further important changes after birth [19], [43].

Only chimpanzees proved evidence for directional asymmetry in the MSP/LSCC angle (right: 82.6±6.4°, left: 78.6±6.7°, p<0.002). In all other instances, we observed a remarkable symmetry (asymmetry ranging from 0.09% to 3.05%).

### Inter-specific comparisons

**Figure 2:**
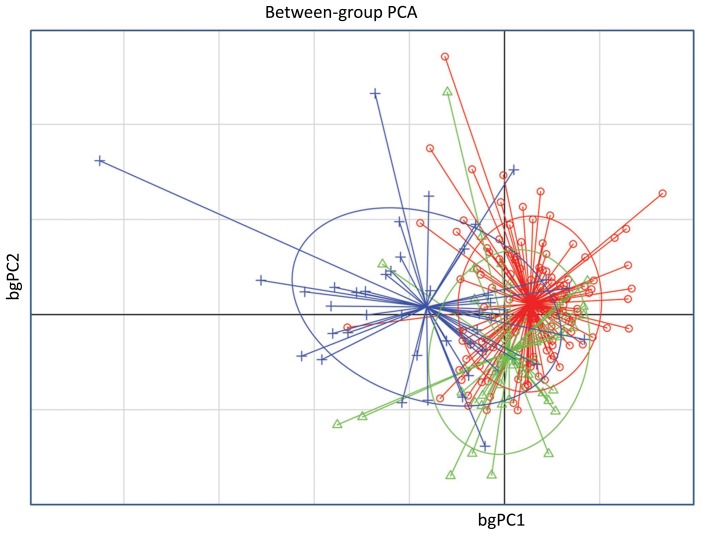
Between group principal component analysis (bgPCA) of the angular measurements. Humans (•) and bonobos (▴) were very closed to each other and distant from chimpanzees (**+**). The ellipses graphically represent the scatter plots encompassing approximately 67% of the subjects. The bgPCA showed a strait overlap of humans with bonobos and a weaker overlap of the latter two species with the chimpanzees.

As shown in Table 4, significant differences were found between chimpanzees and the two other species. Humans and Bonobos shared more similarities. The bgPCA (Figure 2) highlight this result: the chimpanzee group was quite separated from both humans and bonobos, with a weak overlap seen on the graph, while a tighter overlap between humans and bonobos was observed.

Between humans and chimpanzees, MSP/ASCC and MSP/PSCC angles were significantly different (p<0.001), indicating in humans ASCC and PSCC respectively 4.2° and 3.2° closer to the MSP. Thus, the angle between ASCC and PSCC was more open in humans (+5.5°, p<0.001). LSCC was 2.9° more horizontal in chimpanzees (HP/LSCC was 2.9° more open in humans) (p<0.001), 2.8° closer to ASCC in humans (p<0.001), and 8.1° closer to PSCC in chimpanzees (p<0.001). The angle between ASCC and the contra lateral PSCC was 7.6° more open in humans than in chimpanzees (p<0.001). Between chimpanzees and Bonobos, LSCC was more horizontally oriented in chimpanzees (p<0.001). The ASCC/PSCC angle was 3.8° more open in bonobos (p<0.001). PSCC was more closely related to MSP in bonobos (4° closer, p<0.001). The angle between ASCC and the contra lateral PSCC was 5.1° flatter in bonobos (p<0.001). In chimpanzees, as compared to bonobos, LSCC was more horizontally oriented (p<0.001). Only the MSP/ASCC angle exhibited a significant difference (p<0.001) between humans and bonobos.

## Discussion and Conclusions

Differences between humans and *Pan* species were previously noticed in semicircular canal size and shape [19], [35], [44], but not specifically between chimpanzees and bonobos, usually found to be similar (see Table S2). In this regard, we found two significantly distinct groups: humans and bonobos on one side and chimpanzees on the other side. This new finding could have three explanations. First, earlier studies excluded the vestibule from calculations of the SCC planes. Second, the present study used 3D angles instead of projected angles. Finally, previous intra-SCC angle measurements in *Pan* were based on very limited sample sizes. We used the vestibule as it contains the utricule and the saccule that are parts of the vestibular system. The fluids filling the vestibule and SCC play a major role in their physiology by the detection of head rotational movement which contributes to balance and maintaining visual fixation during head movements. Because the utricule and the saccule are parts of this system to detect motion and orientation, their use is functionally relevant in SCC study. The precision and reliability of the vestibule landmark is presented in additional data (Method S1).

A previous bgPCA analysis of overall labyrinth shape revealed significant discrimination among two subspecies of *P. troglodytes* [35] demonstrating subtle, yet significant, differences between *P. troglogytes troglodytes and P. t. verus* and a clear separation between humans and the two chimpanzees groups. However Gunz et al. [35] focused on the total morphological pattern of the labyrinth (through a Geometric Morphometric approach) whereas our study concentrated only on a limited number of angular measurements of the labyrinth. Despite this, our results demonstrate differences between the two species of *Pan* and are in line with Gunz et al. [35] study. We did not find a clear separation between the two subspecies of chimpanzees represented in our sample (*P. t. schweinfurthi* and *P. t. verus*), even though genetical and morphological data suggest that among subspecies of common chimpanzee, *P.t. verus* is the most distinct [24]. As regard the labyrinth, Gunz et al. [35] observed only subtle differences in canal radii. This may explain why our angular values failed to discriminate *P. t. schweinfurthi* and *P. t. verus*.

We found more similarities between humans and bonobos than between chimpanzees and bonobos. The differences recorded between the present study and that of Spoor and Zonneveld (1998) [44] could result from differences in resolution, as the smallest available slice thickness used by the latter (1.5 mm) was higher than our highest available slice thickness (1 mm) (Table S1). Also, we used a sample of *Pan* wild specimens of known geographical origin much larger than in Spoor and Zonneveld (1998) (61 vs 6 *P. paniscus* and 62 vs 7 *P. troglodytes*).

**Figure 3:**
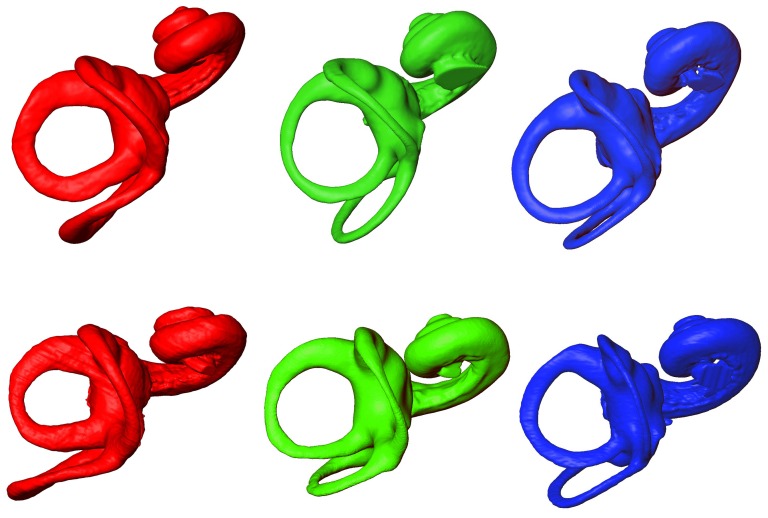
MicroCT-based rendering of the left bony labyrinth superior aspect in: *Homo sapiens* (red, on the left), *Pan paniscus* (green, in the middle) and *Pan troglodytes* (blue, on the right).

**Figure 4:**
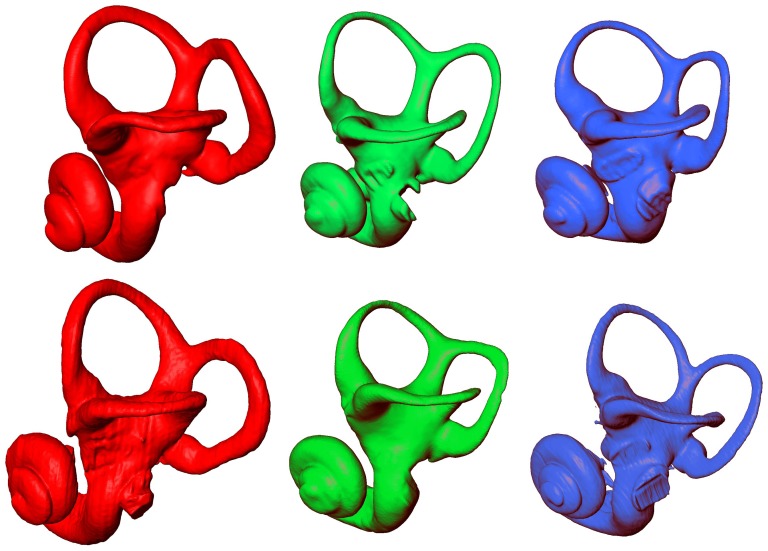
MicroCT-based rendering of the left bony labyrinth lateral aspect in: *Homo sapiens* (red, on the left), *Pan paniscus* (green, in the middle) and *Pan troglodytes* (blue, on the right).

The extent of differences and similarities between *P. paniscus*, *P. troglodytes*, and *H. sapiens* were illustrated in Figure 3 and 4 by the μCT-based reconstruction of 2 humans, 2 chimpanzees and 2 bonobos labyrinths. A simple visual inspection (Figure 3 and 4) showed important intra-specific differences. We remarked morphological differences between *Pan* and humans. The thickness was greater in humans, especially for ASCC and PSCC whereas the 3D curvature did not clearly separate human and *Pan* species. However, these differences did not suffice to identify clear-cut inter-SCC angular inter-specific differences

In the context of our observed significant intra-SCC angular differences between chimpanzees and bonobos, it is important to add that basicranial differences between the two species of *Pan* were demonstrated by Cousin et al. [29] using 83 and 179 specimens of *P. troglodytes* and *P. paniscus* respectively. In their comparative study, the skulls were oriented using the lateral semicircular canal (the so-called «vestibular orientation method»). They found inter-specific differences in basicranial geometry. For some measurements and at each stage of its growth, *H. sapiens* appeared closer in shape to one of the two *Pan* species than they were to each other. Bonobos and humans were closer in the angle between LSCC and a line rejoining Nasion-Vestibule (Na-Op/V). Chimpanzees and humans were closer in the angle between LSCC and Nasion-sella turcica. Even though we could not directly compare our results with those taken from Cousin et al. [29], more studies are needed to investigate in detail, with appropriate comparative samples, how and which labyrinthine features show different orientations when compared to basicranial structures in humans, chimpanzees and bonobos.

We observed that chimpanzees have more horizontally-oriented LSCC. However, to our knowledge, there is currently no satisfactory and consensual explanation based on comparative physiological studies between the *Pan* species and modern humans. There is evidence that the use of bipedality is different between the two species [45]. Using feet contact time and hind limb joint angles, Pontzer et al. [46] suggested that bipedal locomotion might have been relatively costly for bonobos as compared to chimpanzees. This cost may reflect a difference in gait mechanics whereas none of the two *Pan* species is more bipedal than the other [45]. There is still no detailed explanation based on the relationship between locomotor patterns and SCC orientation.

Directional asymmetry was also found in chimpanzees with a more open MSP/LSCC angle on the right side. While gait asymmetry is well documented in chimpanzees with predominance of the right to make ground contact first [47], data are missing on bonobos as well as for bipedality. Our unexpected results for more horizontally oriented LSCC in chimpanzees need to be confirmed by further studies.

The characterization of functional relationships between canal morphology and locomotion is limited by the lack of data, especially in vivo. Only the MSP/ASCC angle clearly discriminates humans from the two *Pan* species. This may be due to the fact that bonobos and chimps show anatomical features that favor versatility [48]. The more open MSP/ASCC angle in *Pan* may contribute to their greater locomotor repertoire, particularly to head rotation through a transverse axis. A second possible explanation may lie in gait modalities of the three species. Bipedal and quadrupedal walking among chimpanzees and bonobos is similar but drastically different from that in humans.

The labyrinth morphology is open to external developmental influences only prior to ossification of the otic capsule, in humans at the end of the second trimester of gestation [28]. No differences were found between MS confirming that SCC orientations undergo no further important changes after birth [19], [43].

Our main result about similarity between bonobos and humans, as compared to chimpanzees, does not fit with our null hypothesis. One explanation is convergent evolution in which bonobos and humans produce independently similar phenotypes due to selection. Such an homoplasic pattern has already been argued for genetic data [2], [49]. Another possibility is convergence following a “random walk” (Brownian motion) evolutionary model (e.g. [50], [51]). Moreover, as demonstrated by complete genome analysis of gorillas, chimpanzees and bonobos [2], [4], incomplete lineage sorting (ILS) [52] may influence phenotypic similarities that humans share with one of these three species but not the others.

Two species might share more similarities in a single trait whereas the phylogenetic tree reveals a different overall pattern of speciation. This might be a consequence of a complex intertwining of genetic diversity from ancestral population, selection and “Brownian walk”. Human and chimpanzee genomes reveals genetic differences accumulated since the divergence of these two species from their common ancestor [5], dating at least to 7–8 million years [6]. The genetically-based estimated time between *P. troglodytes* and *P. paniscus* split is 2.1–1.5 million years [53]. In order to identify the respective role of each process, it would be interesting to investigate the labyrinthine morphology in fossil specimens attributed to *Ardipithecus* (ARA-VP-6/500) [54], and *Sahelanthropus* (TM 266-01-060-1) [55].

A final and more parsimonious explanation is that the bonobo-human labyrinthine shared morphology, more closely represents the *Pan-Homo* ancestral condition with chimpanzees being subsequently derived. Interestingly, the MSP/LSCC angle does not show the same trend as the other angles, showing no significant differences between the three species. This may reflect a primitive shared condition.

The petrous bone is often well preserved in fossil specimens [44], [56]–[59]. However, deformation occurring from compaction and other diagenetic processes [60], [61] makes it sometimes difficult to infer phylogenetic relationships [62] as well as missing data on extant specimens. The remarkable symmetry of the SCC is another important result of this study and may have implications in taphonomy. Further observations on fossil hominids are needed to investigate the potential usefulness of asymmetry to evaluate post-mortem deformation.

Most studies which have attempted to find sexual dimorphism at the inner ear level provided inconsistent results. However our observed sexual dimorphism in ASCC/PSCC and LSCC/PSCC angles are in agreement with results obtained in modern humans either for the bony labyrinth as a whole [63] or on the cochlea only [64], [65].

In conclusion, we have used the largest known sample of individuals to measure the three dimensional orientation of semicircular canals in humans, bonobos and chimpanzees. We have demonstrated in this sample that bonobos SCC orientation is closer to humans than to chimpanzees. This finding may have crucial implications in hominid evolution that still need to be addressed. Additionally, the low intra-individual asymmetrical signal of the inner ear in our sample could open a most interesting track for the study of paleoanthropological records.

## Supporting Information

Table S1 **Detailed information and acquisition modes from the 260 subjects of the present sample.**
(DOCX)

Table S2 **Comparisons with previous studies related to Semicircular Canal orientation.**
(DOCX)

Method S1 **Placement and reliability of the vestibular landmark.**
(DOCX)
