# Supplementary material for: The Human Semicircular Canals Orientation Is More Similar to the Bonobos than to the Chimpanzees
Source: PLoS One. 2014 Apr 7;9(4):e93824. doi: 10.1371/journal.pone.0093824 (PMC3978048; doi:10.1371/journal.pone.0093824)
Supplement: Table S1 — Detailed information and acquisition modes from the 260 subjects of the present sample. (DOCX) [file pone.0093824.s001.docx]

| Reference  Catalog number | Collection* | genus/species | sex | age | MS | scanner | Company | modality | resolution |
| --- | --- | --- | --- | --- | --- | --- | --- | --- | --- |
| M3_F_RG 9338 | MRAC | Pan paniscus | f | u | A1 | Sensation 64 | Siemens | CT | 0.29x0.29x1 |
| A1FMRAC29033 | MRAC | Pan paniscus | f | u | A1 | Esprit | Siemens | CT | 0.39x0.39x1 |
| A1IMRAC9918 | MRAC | Pan paniscus | u | u | A1 | Esprit | Siemens | CT | 0.31x0.31x1 |
| A1IMRAC14738 | MRAC | Pan paniscus | u | u | A1 | Esprit | Siemens | CT | 0.39x0.39x1 |
| A1IMRAC23509 | MRAC | Pan paniscus | u | u | A1 | Esprit | Siemens | CT | 0.39x0.39x1 |
| A1IMRAC88041M2 | MRAC | Pan paniscus | u | u | A1 | Esprit | Siemens | CT | 0.39x0.39x1 |
| A1IMRAC88041M4 | MRAC | Pan paniscus | u | u | A1 | Esprit | Siemens | CT | 0.39x0.39x1 |
| A1MMRAC29047 | MRAC | Pan paniscus | m | u | A1 | Esprit | Siemens | CT | 0.39x0.39x1 |
| A2FMRAC9338 | MRAC | Pan paniscus | f | u | A2 | Esprit | Siemens | CT | 0.36x0.36x1 |
| A2FMRAC11351 | MRAC | Pan paniscus | f | u | A2 | Esprit | Siemens | CT | 0.39x0.39x1 |
| A2FMRAC11352 | MRAC | Pan paniscus | f | u | A2 | Esprit | Siemens | CT | 0.39x0.39x1 |
| A2FMRAC15295 | MRAC | Pan paniscus | f | u | A2 | Esprit | Siemens | CT | 0.39x0.39x1 |
| A2FMRAC15296 | MRAC | Pan paniscus | f | u | A2 | Esprit | Siemens | CT | 0.39x0.39x1 |
| A2FMRAC20882 | MRAC | Pan paniscus | f | u | A2 | Esprit | Siemens | CT | 0.39x0.39x1 |
| A2FMRAC29035 | MRAC | Pan paniscus | f | u | A2 | Esprit | Siemens | CT | 0.39x0.39x1 |
| A2FMRAC29059 | MRAC | Pan paniscus | f | u | A2 | Esprit | Siemens | CT | 0.39x0.39x1 |
| A2FMRAC29060 | MRAC | Pan paniscus | f | u | A2 | Esprit | Siemens | CT | 0.39x0.39x1 |
| A2FMRAC29065 | MRAC | Pan paniscus | f | u | A2 | Esprit | Siemens | CT | 0.39x0.39x1 |
| A2MMRAC11353 | MRAC | Pan paniscus | m | u | A2 | Esprit | Siemens | CT | 0.39x0.39x1 |
| A2MMRAC13202 | MRAC | Pan paniscus | m | u | A2 | Esprit | Siemens | CT | 0.39x0.39x1 |
| A2MMRAC15294 | MRAC | Pan paniscus | m | u | A2 | Esprit | Siemens | CT | 0.39x0.39x1 |
| A2MMRAC29036 | MRAC | Pan paniscus | m | u | A2 | Esprit | Siemens | CT | 0.39x0.39x1 |
| A2MMRAC29064 | MRAC | Pan paniscus | m | u | A2 | Esprit | Siemens | CT | 0.39x0.39x1 |
| J1FMRAC26977 | MRAC | Pan paniscus | f | u | J1 | Xtreme | Scanco | CT | 0.29x0.29x1 |
| J1FMRAC26990 | MRAC | Pan paniscus | f | u | J1 | Xtreme | Scanco | CT | 0.29x0.29x1 |
| J1FMRAC26992 | MRAC | Pan paniscus | f | u | J1 | Xtreme | Scanco | CT | 0.29x0.29x1 |
| J1IMRAC12087 | MRAC | Pan paniscus | u | u | J1 | Xtreme | Scanco | CT | 0.29x0.29x1 |
| J1IMRAC22336 | MRAC | Pan paniscus | u | u | J1 | Xtreme | Scanco | CT | 0.29x0.29x1 |
| J1J2IMRAC23464 | MRAC | Pan paniscus | u | u | J1 | Xtreme | Scanco | CT | 0.29x0.29x1 |
| J1J2MMRAC26938 | MRAC | Pan paniscus | m | u | J1 | Xtreme | Scanco | CT | 0.29x0.29x1 |
| J1J2MMRAC26958 | MRAC | Pan paniscus | m | u | J1 | Xtreme | Scanco | CT | 0.29x0.29x0.5 |
| J1J2MMRAC26979 | MRAC | Pan paniscus | m | u | J1 | Esprit | Siemens | CT | 0.33x0.33x1 |
| J1J2MMRAC26980 | MRAC | Pan paniscus | m | u | J1 | Esprit | Siemens | CT | 0.28x0.28x0.5 |
| J1MMRAC18050 | MRAC | Pan paniscus | m | u | J1 | Esprit | Siemens | CT | 0.29x0.29x0.5 |
| J2FMRAC26936 | MRAC | Pan paniscus | f | u | J2 | Xtreme | Scanco | CT | 0.29x0.29x0.5 |
| J2FMRAC26968 | MRAC | Pan paniscus | f | u | J2 | Esprit | Siemens | CT | 0.35x0.35x1 |
| J2FMRAC29026 | MRAC | Pan paniscus | f | u | J2 | Esprit | Siemens | CT | 0.39x0.39x1 |
| J2IMRAC10198 | MRAC | Pan paniscus | u | u | J2 | Xtreme | Scanco | CT | 0.29x0.29x0.5 |
| J2IMRAC29019 | MRAC | Pan paniscus | u | u | J2 | Xtreme | Scanco | CT | 0.29x0.29x0.5 |
| J2IMRAC29020 | MRAC | Pan paniscus | u | u | J2 | Xtreme | Scanco | CT | 0.29x0.29x0.5 |
| J2IMRAC29021 | MRAC | Pan paniscus | u | u | J2 | Xtreme | Scanco | CT | 0.29x0.29x0.5 |
| J2IMRAC29022 | MRAC | Pan paniscus | u | u | J2 | Xtreme | Scanco | CT | 0.29x0.29x0.5 |
| J2IMRAC29049 | MRAC | Pan paniscus | u | u | J2 | Esprit | Siemens | CT | 0.29x0.29x1 |
| J2J3MMRAC29028 | MRAC | Pan paniscus | m | u | J2 | Esprit | Siemens | CT | 0.39x0.39x1 |
| J2J3MMRAC29056 | MRAC | Pan paniscus | m | u | J2 | Esprit | Siemens | CT | 0.39x0.39x1 |
| J2J3MMRAC29058 | MRAC | Pan paniscus | m | u | J2 | Xtreme | Scanco | CT | 0.29x0.29x0.5 |
| J2MMRAC09369 | MRAC | Pan paniscus | m | u | J2 | Xtreme | Scanco | CT | 0.29x0.29x0.5 |
| J2MMRAC29023 | MRAC | Pan paniscus | m | u | J2 | Xtreme | Scanco | CT | 0.29x0.29x0.5 |
| J3FMRAC26988 | MRAC | Pan paniscus | f | u | J3 | Xtreme | Scanco | CT | 0.29x0.29x0.5 |
| J3FMRAC26993 | MRAC | Pan paniscus | f | u | J3 | Xtreme | Scanco | CT | 0.29x0.29x0.5 |
| J3FMRAC27010 | MRAC | Pan paniscus | f | u | J3 | Esprit | Siemens | CT | 0.35x0.35x1 |
| J3FMRAC29030 | MRAC | Pan paniscus | f | u | J3 | Xtreme | Scanco | CT | 0.29x0.29x0.5 |
| J3IMRAC29027 | MRAC | Pan paniscus | u | u | J3 | Xtreme | Scanco | CT | 0.29x0.29x0.5 |
| J3IMRAC29029 | MRAC | Pan paniscus | u | u | J3 | Xtreme | Scanco | CT | 0.29x0.29x0.5 |
| J3IMRAC29031 | MRAC | Pan paniscus | u | u | J3 | Xtreme | Scanco | CT | 0.29x0.29x0.5 |
| J3IMRAC29032 | MRAC | Pan paniscus | u | u | J3 | Xtreme | Scanco | CT | 0.29x0.29x0.5 |
| J3MMRAC26971 | MRAC | Pan paniscus | m | u | J3 | Xtreme | Scanco | CT | 0.29x0.29x0.5 |
| J2FMRAC26936 | MRAC | Pan paniscus | f | u | J3 | Esprit | Siemens | CT | 0.39x0.39x1 |
| NJ1MRAC20076 | MRAC | Pan paniscus | u | u | NJ1 | Xtreme | Scanco | CT | 0.29x0.29x1 |
| NJ1MRAC84036M6 | MRAC | Pan paniscus | u | u | NJ1 | Xtreme | Scanco | CT | 0.29x0.29x1 |
| NJ1MRAC84036M7 | MRAC | Pan paniscus | u | u | NJ1 | Xtreme | Scanco | CT | 0.27x0.27x1 |
| A1MMRAC29078 | MRAC | Pan troglodytes | m | u | A1 | Esprit | Siemens | CT | 0.39x0.39x1 |
| A1MMRAC83006M16 | MRAC | Pan troglodytes | m | u | A1 | Esprit | Siemens | CT | 0.39x0.39x1 |
| A1FMRAC9931 | MRAC | Pan troglodytes | f | u | A1 | Esprit | Siemens | CT | 0.39x0.39x1 |
| A1FMRAC10448 | MRAC | Pan troglodytes | f | u | A1 | Esprit | Siemens | CT | 0.39x0.39x1 |
| A1IMRAC83006M25 | MRAC | Pan troglodytes | u | u | A1 | Esprit | Siemens | CT | 0.39x0.39x1 |
| A1IMRAC83006M27 | MRAC | Pan troglodytes | u | u | A1 | Esprit | Siemens | CT | 0.39x0.39x1 |
| A1MMRAC10447 | MRAC | Pan troglodytes | m | u | A1 | Esprit | Siemens | CT | 0.41x0.41x1 |
| A1MMRAC11362 | MRAC | Pan troglodytes | m | u | A1 | Esprit | Siemens | CT | 0.39x0.39x1 |
| A1MMRAC29075 | MRAC | Pan troglodytes | m | u | A1 | Esprit | Siemens | CT | 0.39x0.39x1 |
| A1MMRAC29077 | MRAC | Pan troglodytes | m | u | A1 | Esprit | Siemens | CT | 0.39x0.39x1 |
| A2FMRAC8341 | MRAC | Pan troglodytes | f | u | A2 | Esprit | Siemens | CT | 0.39x0.39x1 |
| A2FMRAC8369 | MRAC | Pan troglodytes | f | u | A2 | Esprit | Siemens | CT | 0.41x0.41x1 |
| A2FMRAC9655 | MRAC | Pan troglodytes | f | u | A2 | Esprit | Siemens | CT | 0.41x0.41x1 |
| A2FMRAC83006M34 | MRAC | Pan troglodytes | f | u | A2 | Esprit | Siemens | CT | 0.39x0.39x1 |
| A2IMRAC9728 | MRAC | Pan troglodytes | u | u | A2 | Esprit | Siemens | CT | 0.42x0.42x1 |
| A2IMRAC11762 | MRAC | Pan troglodytes | u | u | A2 | Esprit | Siemens | CT | 0.41x0.41x1 |
| A2IMRAC11987 | MRAC | Pan troglodytes | u | u | A2 | Esprit | Siemens | CT | 0.39x0.39x1 |
| A2IMRAC12014 | MRAC | Pan troglodytes | u | u | A2 | Esprit | Siemens | CT | 0.39x0.39x1 |
| A2MMRAC286 | MRAC | Pan troglodytes | m | u | A2 | Esprit | Siemens | CT | 0.42x0.42x1 |
| A2MMRAC9576 | MRAC | Pan troglodytes | m | u | A2 | Esprit | Siemens | CT | 0.44x0.44x1 |
| A2MMRAC10481 | MRAC | Pan troglodytes | m | u | A2 | Esprit | Siemens | CT | 0.42x0.42x1 |
| A2MMRAC22925 | MRAC | Pan troglodytes | m | u | A2 | Esprit | Siemens | CT | 0.41x0.41x1 |
| A2MMRAC83006M13 | MRAC | Pan troglodytes | m | u | A2 | Esprit | Siemens | CT | 0.39x0.39x1 |
| A2MMRAC83006M17 | MRAC | Pan troglodytes | m | u | A2 | Esprit | Siemens | CT | 0.39x0.39x1 |
| J1J2FMRAC9584 | MRAC | Pan troglodytes | f | u | J1 | Esprit | Siemens | CT | 0.39x0.39x1 |
| J1J2IMRAC11157 | MRAC | Pan troglodytes | u | u | J1 | Esprit | Siemens | CT | 0.39x0.39x1 |
| J1J2IMRAC21700 | MRAC | Pan troglodytes | u | u | J1 | Esprit | Siemens | CT | 0.39x0.39x1 |
| J1MMRAC13718 | MRAC | Pan troglodytes | m | u | J1 | Esprit | Siemens | CT | 0.39x0.39x1 |
| J2FMRAC690 | MRAC | Pan troglodytes | f | u | J2 | Esprit | Siemens | CT | 0.39x0.39x1 |
| J2FMRAC8342 | MRAC | Pan troglodytes | f | u | J2 | Esprit | Siemens | CT | 0.39x0.39x1 |
| J2IMRAC9252 | MRAC | Pan troglodytes | u | u | J2 | Esprit | Siemens | CT | 0.39x0.39x1 |
| J2IMRAC83006M23 | MRAC | Pan troglodytes | u | u | J2 | Esprit | Siemens | CT | 0.39x0.39x1 |
| J2IMRAC83006M24 | MRAC | Pan troglodytes | u | u | J2 | Esprit | Siemens | CT | 0.39x0.39x1 |
| J2IMRAC83006M28 | MRAC | Pan troglodytes | u | u | J2 | Esprit | Siemens | CT | 0.39x0.39x1 |
| J2J3MMRAC559 | MRAC | Pan troglodytes | m | u | J2 | Esprit | Siemens | CT | 0.39x0.39x1 |
| J2MMRAC12185 | MRAC | Pan troglodytes | m | u | J2 | Esprit | Siemens | CT | 0.39x0.39x1 |
| J2MMRAC25491 | MRAC | Pan troglodytes | m | u | J2 | Esprit | Siemens | CT | 0.39x0.39x1 |
| J2MMRAC29076 | MRAC | Pan troglodytes | m | u | J2 | Esprit | Siemens | CT | 0.39x0.39x1 |
| J3FMRAC689 | MRAC | Pan troglodytes | f | u | J3 | Esprit | Siemens | CT | 0.39x0.39x1 |
| J3FMRAC2487 | MRAC | Pan troglodytes | f | u | J3 | Esprit | Siemens | CT | 0.39x0.39x1 |
| J3FMRAC2489 | MRAC | Pan troglodytes | f | u | J3 | Esprit | Siemens | CT | 0.39x0.39x1 |
| J3FMRAC12231 | MRAC | Pan troglodytes | f | u | J3 | Esprit | Siemens | CT | 0.39x0.39x1 |
| J3FMRAC13717 | MRAC | Pan troglodytes | f | u | J3 | Esprit | Siemens | CT | 0.39x0.39x1 |
| J3IMRAC9248 | MRAC | Pan troglodytes | u | u | J3 | Esprit | Siemens | CT | 0.39x0.39x1 |
| J3IMRAC9249 | MRAC | Pan troglodytes | u | u | J3 | Esprit | Siemens | CT | 0.39x0.39x1 |
| J3MMRAC2105 | MRAC | Pan troglodytes | m | u | J3 | Esprit | Siemens | CT | 0.39x0.39x1 |
| J3MMRAC29072 | MRAC | Pan troglodytes | m | u | J3 | Esprit | Siemens | CT | 0.39x0.39x1 |
| MRAC23501 | MRAC | Pan troglodytes | u | u | NJ1 | Esprit | Siemens | CT | 0.39x0.39x1 |
| MRAC91060M406 | MRAC | Pan troglodytes | u | u | NJ1 | Esprit | Siemens | CT | 0.39x0.39x1 |
| MRAC91060M410 | MRAC | Pan troglodytes | u | u | NJ1 | Esprit | Siemens | CT | 0.39x0.39x1 |
| MRAC91060M411 | MRAC | Pan troglodytes | u | u | NJ1 | Esprit | Siemens | CT | 0.39x0.39x1 |
| MRAC830076M15 | MRAC | Pan troglodytes | u | u | NJ1 | Esprit | Siemens | CT | 0.39x0.39x1 |
| NJ1IMRAC10734 | MRAC | Pan troglodytes | u | u | NJ1 | Esprit | Siemens | CT | 0.37x0.37x1 |
| NJ1IMRAC12954 | MRAC | Pan troglodytes | u | u | NJ1 | Esprit | Siemens | CT | 0.39x0.39x1 |
| A1_F_HARV7545 | HARV | Pan troglodytes | f | u | A1 | Volume Zoom | Siemens | CT | 0.46x0.46x1 |
| A1_F_HARV7562 | HARV | Pan troglodytes | f | u | A1 | Volume Zoom | Siemens | CT | 0.42x0.42x1 |
| A1_M_HARV7542 | HARV | Pan troglodytes | m | u | A1 | Volume Zoom | Siemens | CT | 0.39x0.39x1 |
| A1_M_HARV7560 | HARV | Pan troglodytes | m | u | A1 | Volume Zoom | Siemens | CT | 0.44x0.44x1 |
| A1_M_HARV7566 | HARV | Pan troglodytes | m | u | A1 | Volume Zoom | Siemens | CT | 0.47x0.47x1 |
| J1_I_HARV7280 | HARV | Pan troglodytes | u | u | J1 | Volume Zoom | Siemens | CT | 0.39x0.39x1 |
| J2_I_HARV7296 | HARV | Pan troglodytes | u | u | J2 | Volume Zoom | Siemens | CT | 0.39x0.39x1 |
| J3_F_HARV7270 | HARV | Pan troglodytes | f | u | J3 | Volume Zoom | Siemens | CT | 0.41x0.41x1 |
| ADO100 | PAST | Homo sapiens | f | 10.67 | J2 | Mx8000 IDT16 | Philips | CT | 0.49x0.49x0.8 |
| ADO101 | PAST | Homo sapiens | f | 14.08 | J3 | Mx8000 IDT16 | Philips | CT | 0.49x0.49x0.8 |
| ADO102 | PAST | Homo sapiens | f | 14.00 | J3 | Mx8000 IDT16 | Philips | CT | 0.49x0.49x0.8 |
| ADO103 | PAST | Homo sapiens | f | 12.67 | J3 | Mx8000 IDT16 | Philips | CT | 0.49x0.49x0.8 |
| ADO106 | PAST | Homo sapiens | f | 15.50 | J3 | Mx8000 IDT16 | Philips | CT | 0.49x0.49x0.8 |
| ADO111 | PAST | Homo sapiens | f | 12.33 | J3 | Mx8000 IDT16 | Philips | CT | 0.49x0.49x0.8 |
| ADO112 | PAST | Homo sapiens | f | 17.00 | J3 | Mx8000 IDT16 | Philips | CT | 0.49x0.49x0.8 |
| ADO113 | PAST | Homo sapiens | f | 14.67 | J3 | Mx8000 IDT16 | Philips | CT | 0.49x0.49x0.8 |
| ADO114 | PAST | Homo sapiens | f | 13.50 | J3 | Mx8000 IDT16 | Philips | CT | 0.49x0.49x0.8 |
| ADO115 | PAST | Homo sapiens | f | 12.08 | J3 | Mx8000 IDT16 | Philips | CT | 0.49x0.49x0.8 |
| ADO116 | PAST | Homo sapiens | f | 12.92 | J3 | Mx8000 IDT16 | Philips | CT | 0.49x0.49x0.8 |
| ADO117 | PAST | Homo sapiens | f | 14.08 | J3 | Mx8000 IDT16 | Philips | CT | 0.49x0.49x0.8 |
| ADO120 | PAST | Homo sapiens | f | 15.25 | J3 | Mx8000 IDT16 | Philips | CT | 0.49x0.49x0.8 |
| ADO124 | PAST | Homo sapiens | f | 17.17 | J3 | Mx8000 IDT16 | Philips | CT | 0.49x0.49x0.8 |
| ADO128 | PAST | Homo sapiens | f | 15.67 | J3 | Mx8000 IDT16 | Philips | CT | 0.49x0.49x0.8 |
| ADO129 | PAST | Homo sapiens | f | 14.75 | J3 | Mx8000 IDT16 | Philips | CT | 0.49x0.49x0.8 |
| ADO130 | PAST | Homo sapiens | f | 12.92 | J3 | Mx8000 IDT16 | Philips | CT | 0.49x0.49x0.8 |
| ADO131 | PAST | Homo sapiens | f | 12.50 | J3 | Mx8000 IDT16 | Philips | CT | 0.49x0.49x0.8 |
| ADO132 | PAST | Homo sapiens | f | 13.33 | J3 | Mx8000 IDT16 | Philips | CT | 0.49x0.49x0.8 |
| ADO136 | PAST | Homo sapiens | f | 12.00 | J3 | Mx8000 IDT16 | Philips | CT | 0.49x0.49x0.8 |
| ADU060 | PAST | Homo sapiens | f | 26.00 | A2 | Mx8000 IDT16 | Philips | CT | 0.49x0.49x0.8 |
| ADU066 | PAST | Homo sapiens | f | 32.00 | A2 | Mx8000 IDT16 | Philips | CT | 0.49x0.49x0.8 |
| ADU068 | PAST | Homo sapiens | f | 29.92 | A2 | Mx8000 IDT16 | Philips | CT | 0.49x0.49x0.8 |
| ADU069 | PAST | Homo sapiens | f | 21.50 | A1 | Mx8000 IDT16 | Philips | CT | 0.49x0.49x0.8 |
| ADU073 | PAST | Homo sapiens | f | 25.92 | A1 | Mx8000 IDT16 | Philips | CT | 0.49x0.49x0.8 |
| ADU076 | PAST | Homo sapiens | f | 27.75 | A2 | Mx8000 IDT16 | Philips | CT | 0.49x0.49x0.8 |
| ENF090 | PAST | Homo sapiens | f | 10.67 | J2 | Mx8000 IDT16 | Philips | CT | 0.49x0.49x0.8 |
| ENF091 | PAST | Homo sapiens | f | 11.50 | J2 | Mx8000 IDT16 | Philips | CT | 0.49x0.49x0.8 |
| ENF092 | PAST | Homo sapiens | f | 10.50 | J2 | Mx8000 IDT16 | Philips | CT | 0.49x0.49x0.8 |
| ENF097 | PAST | Homo sapiens | f | 9.42 | J2 | Mx8000 IDT16 | Philips | CT | 0.49x0.49x0.8 |
| ENF098 | PAST | Homo sapiens | f | 11.33 | J2 | Mx8000 IDT16 | Philips | CT | 0.49x0.49x0.8 |
| ENF100 | PAST | Homo sapiens | f | 11.17 | J2 | Mx8000 IDT16 | Philips | CT | 0.49x0.49x0.8 |
| ENF101 | PAST | Homo sapiens | f | 6.83 | J2 | Mx8000 IDT16 | Philips | CT | 0.49x0.49x0.8 |
| ENF107 | PAST | Homo sapiens | f | 12.00 | J3 | Mx8000 IDT16 | Philips | CT | 0.49x0.49x0.8 |
| ENF108 | PAST | Homo sapiens | f | 11.75 | J2 | Mx8000 IDT16 | Philips | CT | 0.49x0.49x0.8 |
| ENF109 | PAST | Homo sapiens | f | 8.92 | J2 | Mx8000 IDT16 | Philips | CT | 0.49x0.49x0.8 |
| ENF114 | PAST | Homo sapiens | f | 10.58 | J2 | Mx8000 IDT16 | Philips | CT | 0.49x0.49x0.8 |
| ENF116 | PAST | Homo sapiens | f | 11.50 | J2 | Mx8000 IDT16 | Philips | CT | 0.49x0.49x0.8 |
| ADO138 | PAST | Homo sapiens | f | 12.75 | J3 | Mx8000 IDT16 | Philips | CT | 0.49x0.49x0.8 |
| ADO144 | FMDENT | Homo sapiens | f | 12.25 | J3 | K9500 3D | Kodak | CBCT | 0.3x0.3x0.3 |
| ADO145 | FMDENT | Homo sapiens | f | 14.75 | J3 | K9500 3D | Kodak | CBCT | 0.3x0.3x0.3 |
| ADO148 | FMDENT | Homo sapiens | f | 12.33 | J3 | K9500 3D | Kodak | CBCT | 0.3x0.3x0.3 |
| ADO149 | FMDENT | Homo sapiens | f | 16.08 | J3 | K9500 3D | Kodak | CBCT | 0.3x0.3x0.3 |
| ADO150 | FMDENT | Homo sapiens | f | 14.92 | J3 | K9500 3D | Kodak | CBCT | 0.3x0.3x0.3 |
| ADO152 | FMDENT | Homo sapiens | f | 12.33 | J3 | K9500 3D | Kodak | CBCT | 0.3x0.3x0.3 |
| ADU79 | PAST | Homo sapiens | f | 24.75 | A1 | Brillance 40 | Philips | CT | 0.49x0.49x0.8 |
| ADU80 | PAST | Homo sapiens | f | 42.42 | A1 | Mx8000 IDT16 | Philips | CT | 0.49x0.49x0.8 |
| ADU81 | PAST | Homo sapiens | f | 45.67 | A2 | Mx8000 IDT16 | Philips | CT | 0.49x0.49x0.8 |
| ADU82 | PAST | Homo sapiens | f | 31.92 | A1 | Mx8000 IDT16 | Philips | CT | 0.49x0.49x0.8 |
| ADU85 | PAST | Homo sapiens | f | 38.08 | A2 | Brillance 40 | Philips | CT | 0.49x0.49x0.8 |
| ADU87 | PAST | Homo sapiens | f | 23.58 | A1 | Brillance 40 | Philips | CT | 0.49x0.49x0.8 |
| ADU90 | PAST | Homo sapiens | f | 32.25 | A2 | Mx8000 IDT16 | Philips | CT | 0.49x0.49x0.8 |
| ADU91 | PAST | Homo sapiens | f | 25.42 | A1 | Mx8000 IDT16 | Philips | CT | 0.49x0.49x0.8 |
| ADU92 | PAST | Homo sapiens | f | 32.25 | A1 | Brillance 40 | Philips | CT | 0.49x0.49x0.8 |
| ADU94 | FMDENT | Homo sapiens | f | 22.58 | A1 | K9500 3D | Kodak | CBCT | 0.3x0.3x0.3 |
| ADU95 | FMDENT | Homo sapiens | f | 63.83 | A2 | K9500 3D | Kodak | CBCT | 0.3x0.3x0.3 |
| ADU96 | FMDENT | Homo sapiens | f | 63.50 | A2 | K9500 3D | Kodak | CBCT | 0.3x0.3x0.3 |
| ADU99 | FMDENT | Homo sapiens | f | 26.33 | A1 | K9500 3D | Kodak | CBCT | 0.3x0.3x0.3 |
| ADU100 | FMDENT | Homo sapiens | f | 32.00 | A1 | K9500 3D | Kodak | CBCT | 0.3x0.3x0.3 |
| ADU102 | FMDENT | Homo sapiens | f | 24.08 | A1 | K9500 3D | Kodak | CBCT | 0.3x0.3x0.3 |
| ADU103 | FMDENT | Homo sapiens | f | 18.33 | A1 | K9500 3D | Kodak | CBCT | 0.3x0.3x0.3 |
| ADU109 | FMDENT | Homo sapiens | f | 34.00 | A2 | K9500 3D | Kodak | CBCT | 0.3x0.3x0.3 |
| ADU112 | FMDENT | Homo sapiens | f | 69.33 | A2 | K9500 3D | Kodak | CBCT | 0.3x0.3x0.3 |
| ADU113 | FMDENT | Homo sapiens | f | 43.58 | A2 | K9500 3D | Kodak | CBCT | 0.3x0.3x0.3 |
| ADU115 | FMDENT | Homo sapiens | f | 23.92 | A1 | K9500 3D | Kodak | CBCT | 0.3x0.3x0.3 |
| ADU116 | FMDENT | Homo sapiens | f | 25.67 | A2 | K9500 3D | Kodak | CBCT | 0.3x0.3x0.3 |
| ENF118 | PAST | Homo sapiens | f | 9.75 | J2 | K9500 3D | Kodak | CBCT | 0.3x0.3x0.3 |
| ENF123 | PAST | Homo sapiens | f | 11.67 | J2 | Brillance 40 | Philips | CT | 0.49x0.49x0.8 |
| ENF124 | FMDENT | Homo sapiens | f | 9.58 | J2 | K9500 3D | Kodak | CBCT | 0.3x0.3x0.3 |
| ENF125 | FMDENT | Homo sapiens | f | 2.50 | NJ1 | K9500 3D | Kodak | CBCT | 0.3x0.3x0.3 |
| ADO104 | PAST | Homo sapiens | m | 15.08 | J3 | Mx8000 IDT16 | Philips | CT | 0.49x0.49x0.8 |
| ADO105 | PAST | Homo sapiens | m | 16.67 | J3 | Mx8000 IDT16 | Philips | CT | 0.49x0.49x0.8 |
| ADO107 | PAST | Homo sapiens | m | 14.00 | J3 | Mx8000 IDT16 | Philips | CT | 0.49x0.49x0.8 |
| ADO108 | PAST | Homo sapiens | m | 15.08 | J3 | Mx8000 IDT16 | Philips | CT | 0.49x0.49x0.8 |
| ADO110 | PAST | Homo sapiens | m | 12.17 | J3 | Mx8000 IDT16 | Philips | CT | 0.49x0.49x0.8 |
| ADO119 | PAST | Homo sapiens | m | 12.75 | J3 | Mx8000 IDT16 | Philips | CT | 0.49x0.49x0.8 |
| ADO121 | PAST | Homo sapiens | m | 13.50 | J3 | Mx8000 IDT16 | Philips | CT | 0.49x0.49x0.8 |
| ADO122 | PAST | Homo sapiens | m | 12.92 | J3 | Mx8000 IDT16 | Philips | CT | 0.49x0.49x0.8 |
| ADO123 | PAST | Homo sapiens | m | 12.50 | J3 | Mx8000 IDT16 | Philips | CT | 0.49x0.49x0.8 |
| ADO125 | PAST | Homo sapiens | m | 14.00 | J3 | Mx8000 IDT16 | Philips | CT | 0.49x0.49x0.8 |
| ADO126 | PAST | Homo sapiens | m | 13.42 | J3 | Mx8000 IDT16 | Philips | CT | 0.49x0.49x0.8 |
| ADO127 | PAST | Homo sapiens | m | 12.67 | J3 | Mx8000 IDT16 | Philips | CT | 0.49x0.49x0.8 |
| ADO133 | PAST | Homo sapiens | m | 12.75 | J3 | Mx8000 IDT16 | Philips | CT | 0.49x0.49x0.8 |
| ADO134 | PAST | Homo sapiens | m | 15.08 | J3 | Mx8000 IDT16 | Philips | CT | 0.49x0.49x0.8 |
| ADO135 | PAST | Homo sapiens | m | 13.42 | J3 | Mx8000 IDT16 | Philips | CT | 0.49x0.49x0.8 |
| ADU063 | PAST | Homo sapiens | m | 37.58 | A2 | Mx8000 IDT16 | Philips | CT | 0.49x0.49x0.8 |
| ADU064 | PAST | Homo sapiens | m | 27.58 | A2 | Mx8000 IDT16 | Philips | CT | 0.49x0.49x0.8 |
| ADU070 | PAST | Homo sapiens | m | 37.42 | A2 | Mx8000 IDT16 | Philips | CT | 0.49x0.49x0.8 |
| ADU077 | PAST | Homo sapiens | m | 58.42 | A2 | Mx8000 IDT16 | Philips | CT | 0.49x0.49x0.8 |
| ADU078 | PAST | Homo sapiens | m | 27.08 | A2 | Mx8000 IDT16 | Philips | CT | 0.49x0.49x0.8 |
| ENF093 | PAST | Homo sapiens | m | 11.42 | J2 | Mx8000 IDT16 | Philips | CT | 0.49x0.49x0.8 |
| ENF094 | PAST | Homo sapiens | m | 8.50 | J2 | Mx8000 IDT16 | Philips | CT | 0.49x0.49x0.8 |
| ENF095 | PAST | Homo sapiens | m | 10.67 | J2 | Mx8000 IDT16 | Philips | CT | 0.49x0.49x0.8 |
| ENF096 | PAST | Homo sapiens | m | 9.67 | J2 | Mx8000 IDT16 | Philips | CT | 0.49x0.49x0.8 |
| ENF099 | PAST | Homo sapiens | m | 11.83 | J2 | Mx8000 IDT16 | Philips | CT | 0.49x0.49x0.8 |
| ENF102 | PAST | Homo sapiens | m | 8.33 | J2 | Mx8000 IDT16 | Philips | CT | 0.49x0.49x0.8 |
| ENF103 | PAST | Homo sapiens | m | 10.50 | J2 | Mx8000 IDT16 | Philips | CT | 0.49x0.49x0.8 |
| ENF104 | PAST | Homo sapiens | m | 10.08 | J2 | Mx8000 IDT16 | Philips | CT | 0.49x0.49x0.8 |
| ENF105 | PAST | Homo sapiens | m | 6.25 | J2 | Mx8000 IDT16 | Philips | CT | 0.49x0.49x0.8 |
| ENF106 | PAST | Homo sapiens | m | 9.33 | J2 | Mx8000 IDT16 | Philips | CT | 0.49x0.49x0.8 |
| ENF110 | PAST | Homo sapiens | m | 10.17 | J2 | Mx8000 IDT16 | Philips | CT | 0.49x0.49x0.8 |
| ENF111 | PAST | Homo sapiens | m | 9.83 | J2 | Mx8000 IDT16 | Philips | CT | 0.49x0.49x0.8 |
| ENF112 | PAST | Homo sapiens | m | 10.08 | J2 | Mx8000 IDT16 | Philips | CT | 0.49x0.49x0.8 |
| ENF113 | PAST | Homo sapiens | m | 9.33 | J2 | Mx8000 IDT16 | Philips | CT | 0.49x0.49x0.8 |
| ADO137 | PAST | Homo sapiens | m | 13.92 | J3 | Definition | Siemens | CT | 0.49x0.49x0.75 |
| ADO139 | PAST | Homo sapiens | m | 15.00 | J3 | Mx8000 IDT16 | Philips | CT | 0.49x0.49x0.8 |
| ADO140 | PAST | Homo sapiens | m | 15.75 | J3 | Mx8000 IDT16 | Philips | CT | 0.49x0.49x0.8 |
| ADO141 | PAST | Homo sapiens | m | 12.50 | J3 | Mx8000 IDT16 | Philips | CT | 0.49x0.49x0.8 |
| ADO142 | PAST | Homo sapiens | m | 16.92 | J3 | Brillance 40 | Philips | CT | 0.49x0.49x0.8 |
| ADO143 | PAST | Homo sapiens | m | 16.00 | J3 | Mx8000 IDT16 | Kodak | CT | 0.49x0.49x0.8 |
| ADO146 | FMDENT | Homo sapiens | m | 15.00 | J3 | K9500 3D | Kodak | CBCT | 0.3x0.3x0.3 |
| ADO147 | FMDENT | Homo sapiens | m | 15.17 | J3 | K9500 3D | Kodak | CBCT | 0.3x0.3x0.3 |
| ADU83 | PAST | Homo sapiens | m | 19.08 | A1 | Mx8000 IDT16 | Philips | CT | 0.49x0.49x0.8 |
| ADU84 | PAST | Homo sapiens | m | 41.25 | A2 | Mx8000 IDT16 | Philips | CT | 0.49x0.49x0.8 |
| ADU86 | PAST | Homo sapiens | m | 26.25 | A1 | Definition | Siemens | CT | 0.49x0.49x0.75 |
| ADU88 | PAST | Homo sapiens | m | 38.75 | A2 | Mx8000 IDT16 | Philips | CT | 0.49x0.49x0.8 |
| ADU89 | PAST | Homo sapiens | m | 30.00 | A2 | Mx8000 IDT16 | Philips | CT | 0.49x0.49x0.8 |
| ADU93 | FMDENT | Homo sapiens | m | 26.33 | A2 | K9500 3D | Kodak | CBCT | 0.3x0.3x0.3 |
| ADU97 | FMDENT | Homo sapiens | m | 32.58 | A2 | K9500 3D | Kodak | CBCT | 0.3x0.3x0.3 |
| ADU98 | FMDENT | Homo sapiens | m | 67.17 | A2 | K9500 3D | Kodak | CBCT | 0.3x0.3x0.3 |
| ADU101 | FMDENT | Homo sapiens | m | 58.00 | A2 | K9500 3D | Kodak | CBCT | 0.3x0.3x0.3 |
| ADU104 | FMDENT | Homo sapiens | m | 30.42 | A2 | K9500 3D | Kodak | CBCT | 0.3x0.3x0.3 |
| ADU105 | FMDENT | Homo sapiens | m | 53.33 | A2 | K9500 3D | Kodak | CBCT | 0.3x0.3x0.3 |
| ADU106 | FMDENT | Homo sapiens | m | 34.50 | A2 | K9500 3D | Kodak | CBCT | 0.3x0.3x0.3 |
| ADU107 | FMDENT | Homo sapiens | m | 36.75 | A2 | K9500 3D | Kodak | CBCT | 0.3x0.3x0.3 |
| ADU108 | FMDENT | Homo sapiens | m | 68.67 | A2 | K9500 3D | Kodak | CBCT | 0.3x0.3x0.3 |
| ADU110 | FMDENT | Homo sapiens | m | 66.00 | A2 | K9500 3D | Kodak | CBCT | 0.3x0.3x0.3 |
| ADU111 | FMDENT | Homo sapiens | m | 63.00 | A2 | K9500 3D | Kodak | CBCT | 0.3x0.3x0.3 |
| ADU114 | FMDENT | Homo sapiens | m | 42.00 | A2 | K9500 3D | Kodak | CBCT | 0.3x0.3x0.3 |
| ADU117 | FMDENT | Homo sapiens | m | 36.58 | A2 | K9500 3D | Kodak | CBCT | 0.3x0.3x0.3 |
| ADU118 | FMDENT | Homo sapiens | m | 59.33 | A2 | K9500 3D | Kodak | CBCT | 0.3x0.3x0.3 |
| ENF117 | PAST | Homo sapiens | m | 9.33 | J2 | Mx8000 IDT16 | Philips | CT | 0.49x0.49x0.8 |
| ENF119 | PAST | Homo sapiens | m | 9.33 | J2 | Mx8000 IDT16 | Philips | CT | 0.49x0.49x0.8 |
| ENF120 | PAST | Homo sapiens | m | 11.92 | J2 | Mx8000 IDT16 | Philips | CT | 0.49x0.49x0.8 |
| ENF121 | PAST | Homo sapiens | m | 8.92 | J2 | Mx8000 IDT16 | Philips | CT | 0.49x0.49x0.8 |
| ENF122 | PAST | Homo sapiens | m | 11.92 | J2 | Mx8000 IDT16 | Philips | CT | 0.49x0.49x0.8 |
| ENF126 | FMDENT | Homo sapiens | m | 9.50 | J2 | K9500 3D | Kodak | CBCT | 0.3x0.3x0.3 |

***MRAC**: Musée Royal de l’Afrique Centrale at Tervuren, Belgium. **HARV**: Museum of Comparative Zoology, Harvard University, USA. **FMDEN**: faculty of Dentistry – University of Toulouse, France. **PAST**: Pasteur hospital in Toulouse, France.

**u**: data not available for sex (**f**: Female, **m**: male) and age, MS: maturational stages: **NJ1**: infant (<1 year of age for apes, <3 years of age for humans) with incomplete deciduous dentition; **J1**: infant (1-3 years of age for apes, 3-6 years for humans) with complete deciduous dentition but with no permanent teeth emerged; **J2**: young juvenile (3-6 years of age for apes, 6-12 years for humans) with the second permanent molar not yet emerged; **J3**: old juvenile (6-11 years of age for apes, 12-18 years for humans) with the third permanent molar not yet emerged; **A1**: sub-adult (older than 11 for apes, and 18 for humans) with a complete permanent dentition but unfused spheno-occipital synchondrosis and **A2**: adult (older than 11 for apes, and 18 for humans) with fused spheno-occipital synchondrosis [1].

1. Shea BT (1989) Heterochrony in Human Evolution: The Case for Neoteny Reconsidered. Yearb Phys Anthropol 32: 69-101.
