# Supplementary material for: The Human Semicircular Canals Orientation Is More Similar to the Bonobos than to the Chimpanzees
Source: PLoS One. 2014 Apr 7;9(4):e93824. doi: 10.1371/journal.pone.0093824 (PMC3978048; doi:10.1371/journal.pone.0093824)
Supplement: Table S2 — Comparisons with previous studies related to Semicircular Canal orientation. (DOCX) [file pone.0093824.s002.docx]

| **Authors** | **Species** | **Imaging procedure** | **N** | ASCC/LSCC | ASCC/PSCC | LSCC/PSCC | LSCC right/left | ASCC right /PSCC left | ASCC left/ PSCC right | MSP/ASCC | MSP/LSCC | MSP/PSCC | HP/LSCC |
| --- | --- | --- | --- | --- | --- | --- | --- | --- | --- | --- | --- | --- | --- |
| **Present study** | ***Hs*** | CT scans | **137** | **74.2 ± 4.4°** | **111.2 ± 6.4°** | **88.2 ± 6.2°** | **19.7 ± 8.9°** | **10.6 ± 5.4°** | **11.4 ± 6.4°** | **34.9 ± 5.1°** | **80.9 ± 5.5°** | **145.3 ± 6.2°** | **25.9 ± 6.7°** |
| Blanks et al. (1975)^1^ | *Hs* | Dissection | 10 | 68.2 ± 7.6° | ~ 90° | ~ 90° | 19.8 ± 14.9° | 23.7 ± 6.7° | 24.6 ± 7.2° |  |  |  |  |
| Spoor et al. (1998)^2^ | *Hs* | CT scans | 53 | 68.0 ± 5.0° | 104.0 ± 5.2° | 68.0 ± 5.0° |  |  |  | 36.0 ± 4.9° | 110.0° | 139.0 ± 5.4° |  |
| Hashimoto (2003)^3^ | *Hs* | CT scans | 5 | 89.6 ± 1.8° | 91.0 ± 1.3° | 94.0 ± 3.8° |  |  |  |  |  |  |  |
| Hashimoto (2005)^4^ | *Hs* | CT scans | 7 | 90.1 ± 3.0° | 91.7 ± 1.9° | 94.5 ± 3.3° |  |  |  |  |  |  |  |
| Della Santina et al.  (2005)^5^ | *Hs* | CT scans | 22 | 89.4 ± 6.2° | 94.0 ± 4.0° | 89.6 ± 4.9° | 11.3 ± 6.9° | 15.4 ± 7.3° | 15.2 ± 7.2° |  |  |  |  |
| Ifediba et al. (2007)^6^ | *Hs* | CT scans | 2 | 84.5°/78.8 | 90.5 ± ?° | 88.9°/90.6 |  |  |  |  |  |  |  |
| Cox et al. (2008)^7^ | *Hs* | CT scans | 6 | 85.3 ± 5.8° | 97.1 ± 4.8° | 89.0 ± 6.3° |  |  |  |  |  |  |  |
| Bradshaw et al. (2010)^8^ | *Hs* | CT scans | 20  34 SCC | 85.3 ± 4.4° | 91.4 ± 3.2° | 89.6 ± 5.7° | 11.7 ± 6.3° | 15.9 ± 6.5° | 15.1 ± 6.3° |  |  |  |  |
| Aoki et al. (2012)^9^ | *Hs* | CT scans | 11  22 SCC | 91.5 ± 6.7° | 94.9 ± 3.8° | 91.0 ± 4.9° | 9.8 ± 5.1° | 17.2 ± 9.5° | 19.0 ± 8.8° | 34.5 ± 4.1° | 90.4 ± 2.6° | 128.9 ± 4.7° |  |
| Lee et al. (2013)^10^ | *Hs* | Micro CT scans | 23  40 SCC | 84.4 ± 4.2° | 92.1 ± 3.5° | 86.2 ± 2.4° |  |  |  |  |  |  |  |
| **Present study** | ***Pp*** | CT scans | **61** | **74.8 ± 7.1°** | **109.5 ± 6.1°** | **86.9 ± 10.5°** | **21.3 ± 15.2°** | **12.8 ± 7.8°** | **14.3 ± 9.2°** | **38.1 ± 4.6°** | **82.9 ± 8.6°** | **146.1 ± 7.4°** | **26.1 ± 7.5°** |
| Spoor et al. (1998)^2^ | *Pp* | CT scans | 6 | 55.0 ± 1.9° | 104.0 ± 4.5° | 55.0 ± 1.9° |  |  |  | 38.0 ± 5.5° | 125° | 142.0 ± 6.0° |  |
| **Present study** | ***Pt*** | CT scans | **62** | **77.0 ± 7.4°** | **105.7 ± 7.6°** | **80.1 ± 10.8°** | **22.0 ± 9.5°** | **19.2 ± 9.5°** | **18.0 ± 10.6°** | **39.1 ± 5.9°** | **80.6 ± 6.6°** | **142.1 ± 8.0°** | **23.0 ± 7.2°** |
| Spoor et al. (1998)^2^ | *Pt* | CT scans | 7 | 50.0 ± 3.9° | 102.0 ± 3.9° | 50.0 ± 3.9° |  |  |  | 37.0 ± 2.4° | 128° | 139.0 ± 3.5° |  |

**Table S2.** Review of the previous studies related to SCCs orientation. ***Hs****: Homo sapiens,****Pp****: Pan paniscus,****Pt****: Pan troglodytes*. **N**: Number of studied individuals and sets of SCC of one ear when mentioned.

**^1^** Blanks RH, Curthoys IS, Markham CH (1975) Planear relationship of the semicircular canals in man. *Acta Otolaryngol* 80: 185-196.

**^2^** Spoor F, Zonneveld F (1998) Comparative review of the human bony labyrinth. *Am J Phys Anthropol* 107(27): 211-251.

**^3^** Hashimoto S (2003) Three-dimensional reconstruction and measurement of the human membranous semicircular canal. *Nippon Jibiinkoka Gakkai kaiho* 106(1): 1-6.

**^4^** [Hashimoto S](http://www.ncbi.nlm.nih.gov/pubmed?term=%22Hashimoto%20S%22%5BAuthor%5D), [Naganuma H](http://www.ncbi.nlm.nih.gov/pubmed?term=%22Naganuma%20H%22%5BAuthor%5D), [Tokumasu K](http://www.ncbi.nlm.nih.gov/pubmed?term=%22Tokumasu%20K%22%5BAuthor%5D), [Itoh A](http://www.ncbi.nlm.nih.gov/pubmed?term=%22Itoh%20A%22%5BAuthor%5D), [Okamoto M](http://www.ncbi.nlm.nih.gov/pubmed?term=%22Okamoto%20M%22%5BAuthor%5D) (2005) Three-dimensional reconstruction of the human semicircular canals and measurement of each membranous canal plane defined by Reid's stereotactic coordinates. [*Ann Otol Rhinol Laryngol*](http://www.ncbi.nlm.nih.gov/pubmed/16425559) 114(12): 934-938.

**^5^** Della Santina CC, Potyagaylo V, Migliaccio AA, Minor LB, Carey JP (2005) Orientation of human semicircular canals measured by three-dimentional mutliplanear CT reconstruction. *J Assoc Res Otolaryngol* 6: 191-206.

**^6^** Ifediba M, Rajguru S, Hullar T, Rabbitt R (2007) The role of 3-canal biomechanics in angular motion transduction by the human vestibular labyrinth. *Ann Biomed Eng* 35: 1247-1263.

**^7^** Cox PG, Jeffery N (2008) Geometry of the semicircular canals and extraocular muscles in rodents, lagomorphs, felids and modern humans. *J Anat* 213: 583-596.

**^8^** Bradshaw AP, Curthoys IS, Todd MJ, Magnussen JS, Taubman DS, et al. (2010) A mathematical model of human semicircular canal geometry: a basis for interpreting vestibular physiology. *J Assoc Res Otolaryngol* 11: 145-159.

**^9^** Aoki S, Takei Y, Suzuki K, Masukawa A, Arai Y (2012) Planer orientation of the bilateral semicircular canals in dizzy patients. *Auris Nasus Larynx* 39(5): 451-454.

**^10^** Lee JY, Shin KJ, Kim JN, Yoo JY, Song WC, Koh KS (2013) A morphometric study of the semicircular canals using micro-ct images in three-dimensional reconstruction. *Anat Rec* 296(5): 834-9.
