# Supplementary material for: The Human Semicircular Canals Orientation Is More Similar to the Bonobos than to the Chimpanzees
Source: PLoS One. 2014 Apr 7;9(4):e93824. doi: 10.1371/journal.pone.0093824 (PMC3978048; doi:10.1371/journal.pone.0093824)
Supplement: Method S1 — Placement and reliability of the vestibular landmark. (DOCX) [file pone.0093824.s003.docx]

**Selection and reliability of the vestibular landmark**

Vestibular landmarks have never been used in previous studies. They were of importance in this study as they allow discriminating the geometry of SCCs in the three considered species. The vestibule landmarks were located using a morphological erosion of the volume of the vestibule. First, we closed the vestibule regarding the oval window and the apertures of SCCs and cochlea. Second, we selected the entire volume of the vestibule. Finally we narrowed the volume till becoming a landmark. This procedure was done twice and visually checked on 2D images.

The precision of the vestibular landmarks was assessed by measuring 10 randomly assigned specimens 5 times. These repeats were submitted to geometric morphometric analyses (O’Higgins and Jones, 1998) in order to assess variation due to precision errors. The vestibular landmarks and the 3 type I of Bookstein landmarks (cristagalli, nasopalatine foramen and infraorbital foramina) were placed on the CT images. Then a Principle Component (PC) Analysis of Kendall’s tangent space coordinates was performed using Morphologika ® (O’Higgins and Jones, 1998) to examine the variation between the successive landmarks placements. The procruste analysis was performed without transformation of the landmarks coordinates. PC1 represents more than 93% of the shape variability, PC2 4.6% and PC3 1.1%. The eigenvectors of landmark coordinates are used to calculate the variability of the shape. The proportion of variance accounted for by each PC and the Eigenvectors of the original coordinates on the PCs allows interpreting the PCs in terms of their significance and meaning (Dryden and Mardia 1998). The variability of the landmarks position is calculated from the Norm of landmarks Eigenvector (NE). Maximum precision was obtained by the cristagalli landmark (NE=0.030) followed by the vestibular landmark (NE=0.056) and nasopalatine foramen (NE=0.079). The lowest precision was obtained by the infraorbital foramen (NE=0.67).

Finally, the scatter plot of repeated landmarks placements were compared using the Euclidean distances between the landmarks and their centroid. One tailed Student t-tests were applied to reveal the differences of precision starting from the null hypothesis (H0) that placement precision is different among the four landmarks selected. No significant differences were found between Cristagalli and the vestibule. Both showed a greater precision than nasopalatine foramen (p<0.003) and infraorbital foramina (p<0.001).

In conclusion, the vestibular landmarks showed a good reproducibility in this study, at the level of the most reliable landmarks.

O’Higgins P, Jones N (1998) Facial growth in *Cercocebus torquatus*: an application of three-dimensional geometric morphometric techniques to the study of morphological variation. J Anat 193: 251-272.

Dryden IL, Mardia KV (1998) *Statistical shape analysis.* John Wiley and Sons. London. Facial growth in *Cercocebus torquatus*: an application of three-dimensional geometric morphometric techniques to the study of morphological variation
